# Impact of Japanese Post-Disaster Temporary Housing Areas’ (THAs) Design on Mental and Social Health

**DOI:** 10.3390/ijerph16234757

**Published:** 2019-11-27

**Authors:** Pablo Bris, Félix Bendito

**Affiliations:** Higher Technical School of Engineering and Industrial Design, Technical University of Madrid, Ronda de Valencia, 3, 28012 Madrid, Spain; felix.bendito@upm.es

**Keywords:** temporary housing, *kodokushi*, Japan, disaster, management, mental health

## Abstract

The phenomenon named *kodokushi*, meaning death alone without the care or company of anyone inside temporary housing, appeared after the Kobe earthquake in Japan in 1995 with some 250 cases. This paper analyzes the evolution of Japanese temporary houses—to attempt to prevent the problem of *kodokushi*—from the point of view of management, how services and activities are organized, and design. We will use case studies as our methodological tool, analyzing the responses in 1995 Kobe (50,000 THs), 2004 Chūetsu (3000 THs), 2011 Tōhoku (50,000 THs), and 2016 Kumamoto (4000 THs). This article shows how the Japanese THAs follow a single design that has undergone very little variation in the last 25 years, a design which promotes the social isolation of their residents, making recovery—from the psychological perspective—and helping the most vulnerable members of society, more difficult. In small scale disasters (Chūetsu) applying organization and management measures was able to correct the problems caused by design and there were no cases of *kodokushi*: in large-scale disasters (Tōhoku), however, the difficulties to implement the same measures resulted in the reappearance of new cases at rates similar to Kobe’s. Our main conclusion is that the design of Japanese THAs must be reconsidered and changed to respond to the real needs of the most vulnerable groups.

## 1. Introduction

Japan is one of the most seismically active countries in the world, with more than 2000 fault lines. From 2004 to 2013, 18.5% of the earthquakes in the world with a magnitude equal to or greater than six on the Richter scale took place in Japan [1]. In line with its extremely high exposure to risk and the country’s economic level, Japan has the most advanced and widespread disaster forecasting, measurement, and public education system in the world to channel end-to-end response and management [2].

### 1.1. Importance of Shelter and Housing after a Disaster

One of the most visible consequences of disaster is when a large number of houses are destroyed or rendered uninhabitable and a large number of people find themselves homeless and in need of a safe place to live. As a part of disaster management, shelter-after-disaster management attempts to respond to this need during the emergency phase and afterwards during recovery work. However, as Ashdown points out [3], “Providing adequate shelter is one of the most intractable problems in international humanitarian response.”

### 1.2. Introduction to Temporary Housing (TH)

The purpose of temporary solutions is to support victims during the recovery period [4]. However, unlike temporary or emergency shelter, focused on providing shelter for the period immediately after the catastrophe and when life is interrupted, TH allows inhabitants to return to their domestic responsibilities and daily routines [5]. When people lose their houses, they lose more than just a place to live. They also lose their privacy, their identity, and even the recognition of their dignity as people [6]. TH are not deployed just to decrease the vulnerability of the victims to disease, health problems or future disastrous events but to provide a space able to guarantee appropriate conditions of protection, habitability, dignity, and privacy [7]. Temporary housing is a determining factor in recovery. It is a support for family and community life enabling people to make a living, maintain families, and move and communicate freely [8]. While the victims lack safe and decent housing, recovery work cannot be done efficiently [9], which will feed back into a worsening of the social and economic impact of the disaster [4,10]. Having appropriate housing is a first step towards creating a certain sense of normalcy in the life of affected communities [11].

### 1.3. Industrialized Temporary Housing

To respond to the urgent demand for large amounts of temporary housing in a short period of time, the most frequent option is to leverage on the advantages provided by industrialized construction in terms of efficiency and production capacity. Prefabricated housing offers high availability, quick and easy installation, building quality, sufficient comfort and durability, and the possibilities of disassembly and portability [12]. However, those systems are associated with a series of logistics and design problems [11]. The most common criticisms are related to aspects like descontextualization, lack of adaptability, cultural unsuitability, the imposition of standards, consumption of economic resources, excessively prolonged stays therein, environmental stress, lack of consideration for local resources, and, in brief, temporary housing’s disconnect from the rest of the recovery process when TA is understood as a product [13,14,15,16,17].

### 1.4. Temporary Housing in Japan

The post-disaster shelter management model in Japan is distributed along three stages or phases ([18], p. 194): Emergency shelter (or evacuation center in Japan), which may be used for days, weeks, or at most months; provisional housing, whose use is variable, since it depends on the time needed to rebuild definitive housing, but can be from one to five or six years; and permanent housing.

Over the last few years, different alternatives have been posed to cover the second stage of provisional housing: temporary housing, new housing, prefabricated housing assembled after the disaster; private apartment rentals; existing public housing; and old government housing previously used by government officials ([18], p. 194). However, this article limits itself to the management, organization, and, fundamentally, the design of temporary housing—and of the camps where they are set up—in the latest disasters to have occurred in Japan. It does not question which of the options from this intermediate phase are better, since temporary housing has been demonstrated to be the indisputable best alternative after large-scale disasters. In the last two (Kobe in 1995 and Tōhoku in 2011) 50,000 units were manufactured in each case.

### 1.5. Temporary Housing and THA Management in Japan

The regulations related to managing disasters in Japan has been progressively adjusted over the last century with input from lessons learned in the innumerable disasters to have struck the island country.

In Japan’s recent history, the Great Kanto earthquake, a 7.9 on the Richter scale, of 1 September 1923, is a benchmark for the use of temporary housing. The destruction of more than 300,000 houses was a direct consequence of the earthquake but mainly as a result of the fires afterwards. Tokyo’s parks played a key role [19] for the installation of the more than 170,000 barracks which had been built, according to metropolitan police, in just six months after the quake, by February 1924 ([20], Table 3.8). Owing to this earthquake, the Japanese government developed “The Imperial Reconstruction Plan,” which is the basis for its current structure ([21], p. 20).

The supply of temporary housing after a disaster is regulated by the Disaster Relief Act of 1947, drafted after the Showa Nankai earthquake, with an 8.0 on the Richter scale [22]. The standards that the housing must comply with were dictated the Ministry of Health, Labor, and Welfare until 2013, after which they were transferred to the Cabinet Office ([20], p. 4.34). The common legislation is national, as well as the funding, which is provided by the central government. Management of location, manufacturing, and assembly of temporary housing is entrusted to prefectures, which may transfer it to the larger municipalities.

Since the disaster of the Great Niigata Fire in 1955, temporary housing has been prefabricated [23]. All of the prefectures have agreements with the Japan Prefabricated Construction Suppliers and Manufacturers Association (JPA). Thanks to these agreements, the construction and assembly of temporary housing is relatively quick, since standards are already predefined by the state. Even so, since they report to different prefectures, the quality and standards of the temporary housing may vary in the same disaster situation. Delays implementing temporary housing complexes can most often be attributed to difficulties finding space to set up the camps. Government policy requires the land to be public property and safe in the event of future disasters ([18], p. 197).

Japan’s management of temporary housing is among the most sophisticated in the world. Over the last 25 years, it has demonstrated this prowess in two large-scale disasters: the Hanshin Earthquake in 17 January 1995, with a 7.3 on the Richter scale, which especially affected the city of Kobe, and the more recent Great East Japan Earthquake, on 11 March 2011, a complex disaster that included a 9.0 Richter magnitude earthquake, the tsunami caused afterwards, and due to the tsunami, the Fukushima nuclear accident. In both cases, temporary housing programs were launched that meant the manufacture of nearly 50,000 units (48,000 in the first case and 48,700 in the second).

### 1.6. Kodokushi or Lonely Death

These two last disasters have a close relationship from the point of view of temporary housing since the management of the disaster of 2011 Tōhoku was built on the lessons learned after the disaster of 1995 Kobe. The main symptom of the poor organization of the TH in Kobe was the appearance of a phenomenon unknown at that time in the field of temporary housing: *kodokushi* or lonely death, when a person dies alone in their home without the care or company of another person. Their body may be found days, weeks, and even months later, and it is not a phenomenon limited to THAs (Temporary Housing Areas). Although there is no official national statistics, there are around 32,000 kodokushi cases in Japan every year [24]. These cases are related to the aging of the population and the growing tendency to live alone [24,25,26]. Nevertheless, in Kobe residents of the THAs “become solitary not just for reasons of age and infirmity but also because the residences did not provide significant opportunities to socialize or build ties for the dislocated refugees” ([25], p. 220). That is to say, the conditions that can cause kodokushi among the most vulnerable affected people were generated because they were moved from their original homes–where they had a network of social ties-to the THAs.

The Japanese government did not have information about the cases of *kodokushi* in any of the THAs before Kobe but up to 230 cases occurred in the Kobe TH the five years they were in operation.

The World Health Organization (WHO) ([27], p. 100) defines health as the “state of complete physical, mental and social well-being instead of the mere absence of disease or infirmity.” The distinction between physical, mental, and social health [28] has been also used in disaster literature.

The changes applied to disaster management after Kobe focused on trying to prevent *kodokushi* through a variety of initiatives [18,26,29,30]. First, initiatives were devised to directly support the mental health of residents by establishing medical and psychological support services in THAs. Second, initiatives were rolled out to improve the social health of residents: moving pre-disaster communities together to the same camp and place THAs as close as possible to the residents’ original houses as well as organizing activities in the THs to strengthen community bonds.

In the first major disaster after Kobe, Chūetsu 2004, 6.8 on the Richter scale, 3460 temporary houses set up, all of the abovementioned measures were implemented. The result was that for three years in which the THAs were operating, there was no cases of *kodokushi* ([30], p. 37).

After the Tōhoku earthquake, an attempt was made to implement the same measures as in Chūetsu: medical and care services were rolled out in the THAs and activities were organized to improve community bonds. However, due to the magnitude and complexity of the disaster, guaranteeing that the pre-disaster community was placed in the same THA and that their location was close to the affected areas was not always a possibility. There were 230 cases of *kodokushi* in the THAs in Tōhoku in six years [31]. Despite their best efforts, the figures were very similar to those in the Kobe disaster.

### 1.7. Goal of This Paper and Main Conclusions

The goal of this paper is twofold: to determine the reason the measures to eliminate the problem of *kodokushi* in the THAs in 2011 Tōhoku failed when they were able to eliminate it in the 2004 Chūetsu disaster and to learn from the latest disasters to improve the design of the THAs to prepare for the next large-scale disaster that hits Japan.

The studies on the mental health of residents in THAs usually employs a sociological or medical approach but practically no study, with the exception of Smith [32], bears in mind the effect of the design of the THAs—which determines very specific lifestyles—on residents’ mental health.

The main conclusion of the study is that Japanese THAs have not changed their design in terms of equipment, size, type, etc., in the last 25 years. All of the measures adopted since 1995 Kobe to reduce or eliminate *kodokushi* have been related to their management (tweaks to the entry system or placing the THAs as close as possible to the affected people’s original houses) and the activities and services organized inside the camps. However, the THA design promotes a lifestyle that encourages residents’ independence and self-sufficiency but also their isolation, a lifestyle that makes it harder to help the most vulnerable residents from the psychological point of view. When the disaster is small, like in Chūetsu, applying appropriate management policies (putting THAs close to affected areas and maintaining the communities that existed prior to the disaster together in the THAs) in concert with the medical and psychological support services and activities to improve community bonds was able to eliminate *kodokushi*.

However, in a large-scale disaster like in Tōhoku, in which ensuring the application of the management measures was impossible, organizing and instituting activities and services was not enough to prevent *kodokushi*, which reached levels on par with Kobe.

The architecture and design of the THAs, far from a solution, has become a part of the problem. Our proposal is to reassess THA design to promote lifestyles that advance not only physical health but also mental and social health.

## 2. Materials and Methods

Case studies will be our methodological research tool. This method has demonstrated its validity in the social science disciplines and in public policy analysis [33,34]. Our case study analyzes in-the-field management of temporary housing during the main disasters that have struck Japan since the Kobe earthquake in 1995. These are 1995 Kobe, 2004 Chūetsu, 2011 Tōhoku, and 2016 Kumamoto, and its relationship with the kodokushi phenomenon.

The case studies are based on data from secondary sources published in English (literature on the management of the disasters studied) and primary sources (official Japanese government documents). To gather the specific information on design and architecture (more graphic), we turned to secondary sources in English and Japanese, from existing literature and the documentation published by construction companies and from architecture, design, and urban development magazines and publications. To obtain the data on the recorded cases of *kodokushi* we used research and focused on press clippings since the Japanese government does not publish statistics on these kinds of cases.

The data collected are both quantitative and qualitative. The first ones, on which existing literature has placed emphasis, are date from the corresponding prefecture’s management regarding: how many houses are built, the companies in charge, the manufacturing and assembly times, how long camps are used, how close the THAs are to the original houses destroyed or damaged, the criteria to enter the THAs, and the percentage of THs with respect to other provisional housing (like private home rentals and public housing). Data on *kodokushi* cases registered in THA in each of the four disasters are also included. The second ones, which literature has generally left by the wayside or analyzed only briefly, are data related to the organization inside the THAs (by the Japanese government, NGOs, private organizations, etc.) of activities and services like medical and psychological care, day centers to care for the most vulnerable residents, social activities in community centers, and workshops, etc.

Given the nature and objectives of the investigation, we will perform a qualitative analysis of the data that, once collected and classified by common themes to each case study, will be put in relation by comparing the different data sets within and between cases. Conclusions will be constructed from the interpretation of the results, with the objective of developing appropriate policies and practical strategies based on the real needs of the population.

## 3. Case Studies

### 3.1. Kobe 1995

The Hanshin Earthquake (7.3 on the Richter scale) of 17 January 1995 mainly affected the city of Kobe, in the prefecture of Hyogo. It caused 6000 deaths, destroyed more than 100,000 and damaged more than 140,000 houses, and displaced more than 300,000 people [20]. More than 390,000 people lost their houses as a result [35].

#### 3.1.1. Management and Organization

The government of the prefecture of Hyogo was in charge of building the THs. More than 48,000 houses were manufactured, built by 21 different companies. Due to the large number of THs, in addition to the 14 companies that belonged to the JPA, another seven companies were involved ([20], p. 3.13). Construction proceeded at a good pace and by the end of March, just two months after the disaster, the first 30,000 units were already ready. In August 1995 (nearly seven months after the disaster), all the THs that had been manufactured were assembled. The delay of the last 18,000 units was due to difficulties finding land and not to manufacturing issues [26]. Kobe’s THAs were used for five years.

Most of the housing destroyed by the earthquake were wooden houses downtown, which meant there was a lack of areas available near the center to set up the THAs. More than 50% of the housing built were placed on the outskirts of the city (on the man-made Port Island, Rokkö Island, and beyond Rokkö Island), in hard-to-reach areas far from the affected housing. To not have to go to the camps, some of the affected people extended their stays at the emergency shelters that were near the affected areas to up to a year and, as a result, up to 5000 temporary houses were still empty at the end of 1995 ([35], pp. 60–61).

Entry into the THAs was decided by lottery, without considering the pre-disaster communities [29]. The Japanese system [36] prioritized entrance to the THAs for those affected people who could not afford a house, starting with the elderly and disabled, meaning that in Kobe’s THAs 30% of residents were seniors and disabled, in comparison to 14% in the overall population [35]. Although these measures were designed to help the most disadvantaged segments of the population recover [37], they had the opposite effect. The THAs furthest from downtown were the first to be built (since the land was publicly owned) and it was there that the most disadvantaged entered first, leaving them isolated in the outer districts ([26], VII, 21).

Until the Kobe earthquake, the Japanese system was characterized by its single-track approach [29]: its provisional housing supply was covered exclusively through THAs.

On this occasion, in some of the THAs, community centers were opened in which neighbors helped by volunteers organized activities to strengthen relationships ([26], VII, 22).

#### 3.1.2. Design

Kobe’s THs used light metallic-structure prefab, over which elevated flooring made of plywood sheets was placed 30 cm above the ground. The ceiling and walls were composed of thin wooden sheet plated by thin galvanized iron. There was no thermal insulation or any type of noise cancellation. In some exceptional cases, air conditioning units were installed but most of the residents did not have means to pay for them so they were used only rarely ([26], VII, 22).

**Figure 1:**
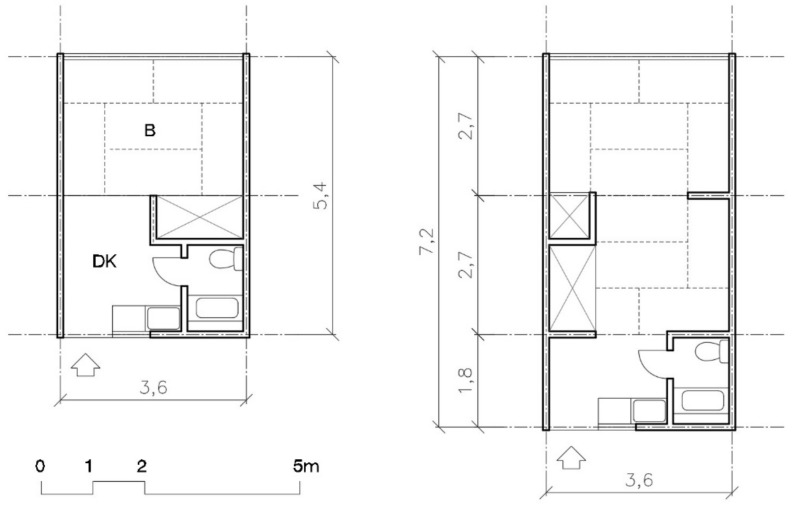
Plan of Kobe’s TH models: one (20 m^2^) and two bedrooms (26 m^2^) [38].

Two models were offered: one bedroom with 20 m^2^ and two bedrooms with 26 m^2^. All the houses were equipped with a prefabricated bathroom and a small kitchen. The rooms were sized using tatami mats (0.9 × 1.8 m), which determined the depth of the room—2.7 m—and the house’s total width—3.6 m (see Figure 1).

**Figure 2:**
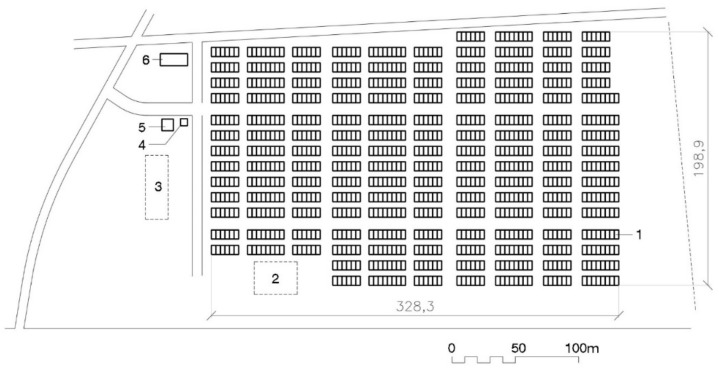
Plan of Kobe’s 1000 THA. 1—Temporary housing row; 2—Playground; 3—Plantation; 4—Nursery; 5—Community Center; 6—Contact Center [39].

Most of the THAs could hold 250 to 1000 temporary houses, although there were exceptions on both ends of the spectrum, including a camp with more than 2000 houses ([26], VII, 22). The total surface area of the THAs was approximately 100 m^2^ for each temporary housing module. Layout was quite similar: matrices of rows of eight to ten houses facing the same way (see Figure 2). Parking places were located inside and the community centers which were built in some of the THAs (the biggest one) on the perimeter, between the parking and the housing.

#### 3.1.3. Kodokushi

According to Ueno Yasuhiro, assistant professor of forensic medicine at the Medical Department of Kobe University, there were 253 cases of *kodokushi* in the five years the Kobe THAs were in use, from 9 March 1995 to 5 May 1999. The prefecture of Hyogo, based on information in the news, recorded 238 cases ([26], IV, 8).

### 3.2. Chūetsu 2004

The Chūetsu earthquake (6.8 on the Richter scale) on 23 October 2004, mainly affected the prefecture of Niigata. It caused 68 deaths, destroyed more than 3175 and partially affected more than 13,810 houses [20].

#### 3.2.1. Management and Organization

A total of 3460 temporary houses were built. Since the area is mountainous and winter was imminent, focus was placed on preparing the houses for snow. In two months (25 December) all the houses were completed, distributed over 64 THAs in 13 different municipalities [20]. The THs were used for three years; in December 2007 the last THA was closed ([40], p. 71).

The land for the THAs was chosen based on proximity to affected housing to enable people to continue with their normal lives: same schools, places of work, businesses, and activities as before the disaster ([20], p. 3.21). When publicly-owned land near affected areas could not be found, the problem was resolved by neighbors’ voluntary transfer of privately-held plots ([23], p. 8).

The Kobe lottery system was avoided and maintaining original communities in the same THAs was prioritized [20,29,30,40].

Provisional housing in this case was not reduced to THAs and aid was offered to rent private houses and the public housing available was used. However, all these alternatives were rare and symbolic in comparison to the use of new TH units ([40], p. 70).

Inside the THAs, welfare facilities and daycare services were built for the residents to offer seniors food and bath services ([40], p. 72). Workshops were also held to improve and customize the porches on the front of TH units, organized by professors from Niigata University through the project “Temporary open café” [30].

#### 3.2.2. Design

The TH construction system was similar to the one used in Kobe (light metallic prefab) but a few improvements were made like thermal insulation and the generalized installation of air conditioning units. Advice was also distributed amongst residents to reduce, collect, and remove condensation and mildew inside the houses.

**Figure 3:**
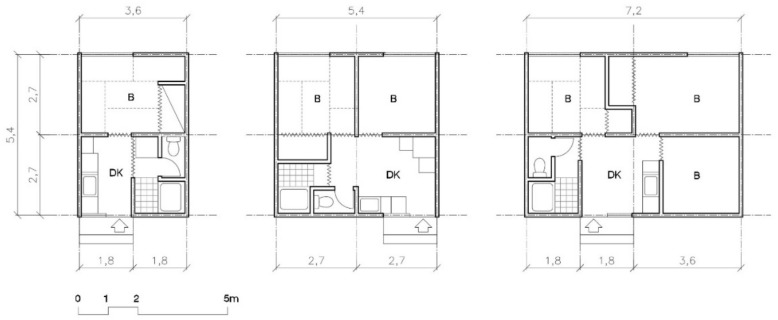
Plan of Chuetsu, Tohoku, and Kumamoto’s TH models: one (20 m^2^), two (30 m^2^) and three bedrooms (40 m^2^) ([30], p. 34).

Three models were offered, one (20 m^2^), two (30 m^2^), and three (40 m^2^) bedroom. The layout was also changed: all three had the same width (5.4 m) so all the rooms could have natural lighting (see Figure 3).

**Figure 4:**
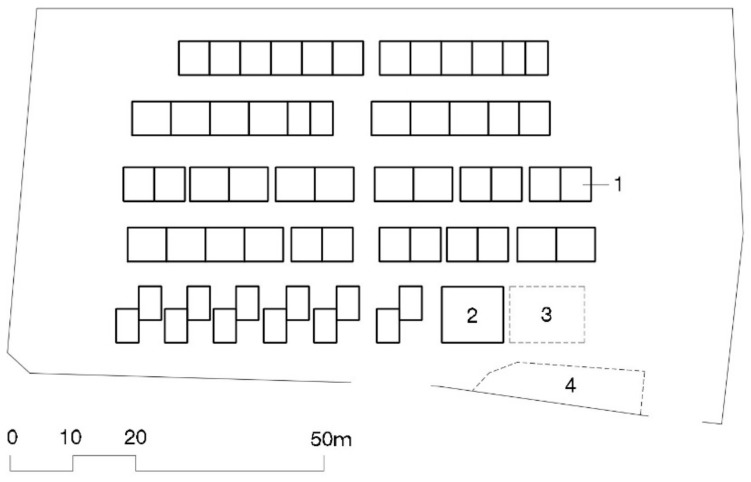
Plan of Chuetsu’s 59 THA. 1—Temporary housing row; 2—Community Center; 3—Playground; 4—Parking area [41].

The THAs were much smaller than in Kobe, from 20 to 250 TH modules ([40], p. 73). The surface size per house in these camps was kept at 100 m^2^. Layout was very similar to Kobe’s with matrices of rows facing the same direction, this time with fewer units, two to six. Community centers were included, one for every 50 THs [29,30], which were placed at the entry to the THAs next to the parking lot (see Figure 4).

#### 3.2.3. Kodokushi

No cases of *kodokushi* were recorded in Chūetsu during the three years the THAs were open and in operation [30].

### 3.3. Tōhoku 2011

The Great East Japan Earthquake (9 on the Richter scale, the strongest in the history of Japan and the fourth biggest in the world since 1900), on 11 March 2011, affected a huge number of different sized communities in three prefectures, Iwate, Miyagi, and Fukushima, all in the region of Tōhoku. It provoked more than 15,000 deaths, destroyed more than 129,000 and damaged more than 266,000 houses. It was a complex disaster. The earthquake set off a tsunami that flooded and devastated 56,100 hectares and caused a nuclear accident at the Fukushima plant, which reached a radioactive contamination of 7, so 90,000 residents in the areas near the affected nuclear plant had to be evacuated [20].

#### 3.3.1. Management and Organization

The extension of the catastrophe’s area, as well as the specificity of the location of the affected areas due to the radioactive contamination from Fukushima, resulted in major differences in how the different THAs were managed ([18], p. 195). Here we will focus on the most widespread responses and not on exceptional cases.

Like in previous disasters, all the prefectures had agreements with the JPA prefabrication companies who were the ones to manufacture most of the units ([18], p. 195): some 43,000 ([20], p. 7.18). However, given the magnitude of the catastrophe (more than 50,000 temporary houses were manufactured), other companies had to be contracted, like members of the Japan Federation of Home Production Organizations and the Japan Wooden Housing Industry Association [42]. The latter wooden manufacturing companies built more than 6000 units, 4000 of which in Fukushima ([18], p. 194). It took approximately two months to manufacture the prefab housing, and the wooden houses a bit more. However, it took several more months for many of the THAs to become available, especially along the northern coast of Sendai, where the lack of available land resulted in delays opening the THAs ([18], p. 196). In September 2018 —7 and a half years after the earthquake—more than 5600 people were still living in the THAs in Iwate, Miyagi, and Fukushima [43].

In Tōhoku, like in Sichuan, as a general policy an attempt was made to set up the THAs near the affected houses [29]. However, the complexity and scale of the disaster meant it was not possible in many cases: the radiation around Fukushima triggered general evacuations, including for those who had lost their houses, of populations that had to go to provisional housing further away. In the areas affected by the tsunami it was hard to find plots to set up the THAs on, so those affected had to move to areas far from their original houses ([23], p. 10).

The difficulties finding safe places for the THAs and the existence of very little initial offering of spaces in the few that were open brought about the return of the lottery system used in Kobe. In some cases, the original community was able to be transferred together to the same THA [29], and there are studies that document these strategies, called community-based THAs [23]. However, the trouble finding safe places for the THAs meant the supply of THs for displaced persons in the emergency camps fell far short of demand. In many cases, a lottery system was used to enter the available THAs ([18], p. 198).

In Tōhoku, the supply of provisional housing was extended considerably: in August 2012 the more than 48,000 temporary houses that were manufactured, more than 19,000 public houses, and more than 67,000 private rentals were occupied ([20], p. 4.35). The distribution of the three types was not uniform over the three prefectures: in Iwate and in northern Miyagi almost only prefab housing was installed, while in the rest of Miyagi and in Fukushima mostly private rental apartments were used ([18], p. 195).

Just like in Chūetsu, a community center was built for every 50 TH to organize activities to improve and encourage relationships between residents ([20], p. 4.36). The activities were organized by different stakeholders: volunteers, NGOs, resident associations [18] (p. 197). Local government civil servants drove the creation of neighborhood associations in the THAs. These associations were mostly created in the community-based THAs ([23], p. 11). Like in Chūetsu, centers to care for the elderly were also included in some THAs. Local governments organized patrols in collaboration with a number of NGOs to prevent resident isolation and cases of *kodokushi*. These patrols visited the most vulnerable residents’ houses one by one to offer them psychological support ([32], p. 168). Another of the initiatives was a Meals-on-Wheels service, which consisted of pre-cooked food trucks which visited the THAs regularly. They parked inside the THAs and set up a little stand with chairs, tables, and music ([32], p. 166).

Besides the general activities described above, implemented in most of the THAs in Tōhoku, other special initiatives were also rolled out like the “Do it Yourself” and “Home for All” projects, the former in the Tagajo City THA, in the Miyagi prefecture, and the latter in up to 16 THAs in areas affected by the tsunami. The “Do it Yourself” project, which began in December 2011, is a collaboration between two private companies, the Royal Home Center Co. Ltd. (a home improvement business) and Daiwa House Industry (one of the TH manufacturers in the JPA), who provided materials and advice so residents could improve and customize their houses by building rims, benches, and fences [44]. The “Home for All” project, which began in October 2011, is an initiative of the architect Toyo Ito, who suggested building centers for meetings and celebrations through donations and collaborating with other local Japanese architects. The project’s main feature is that residents participate in the construction as a community-building strategy. The architecture is also adapted to the local context and the use of the building according to the residents’ needs [45].

#### 3.3.2. Design

Most of the housing built used light metallic structure prefab and were very similar in their construction and look and feel to those in Chūetsu. There was a much lower percentage (12%) albeit a large number (around 6000) of houses that were built with wood, in a vast array of construction styles, since in Fukushima alone 26 different companies worked on building these types of houses [46].

Because of the large amount of prefab houses that needed to be built, the use of minimum standards, and the tight deadlines for assembly the building quality was very low [29] and a variety of problems arose in assembly like gaps between walls and roofs, drafts and design, like lack of shelves and storage areas, places to sit outside, lack of an anwing or enclosure and veranda at the entrance door. In interviews with residents, complaints about the lack of ventilation under the floor (resulting in mildew), condensation on windows, the impossibility of dissipating heat in the summer, insufficient insulation, and noise were all recorded [42].

Housing built with wood, although the manufacturing and construction period was longer than for prefab, had a similar price and were very well received ([20], p. 4.36).

**Figure 5:**
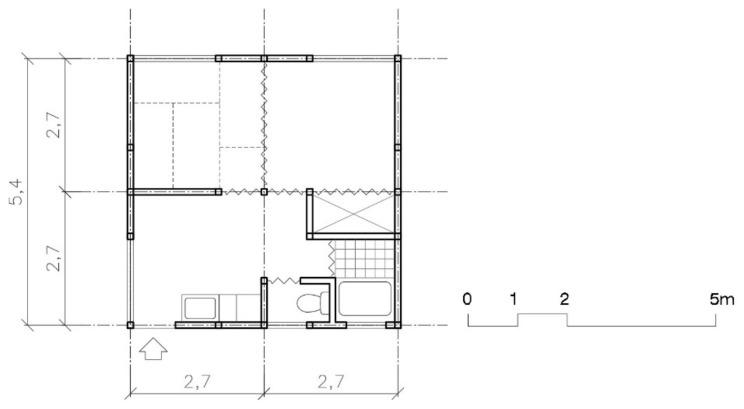
Plan of a Tohoku wooden two bedroom model (30 m^2^) [47].

Like in Chūetsu, in general three models were offered, one (20 m^2^), two (30 m^2^), and three (40 m^2^) bedroom. The layout was very similar, including the layout for the models built with wood (see Figure 5). Exceptionally, in the wooden models a larger model (four bedrooms) was offered for the larger families evacuated out of Fukushima ([18], p. 195).

**Figure 6:**
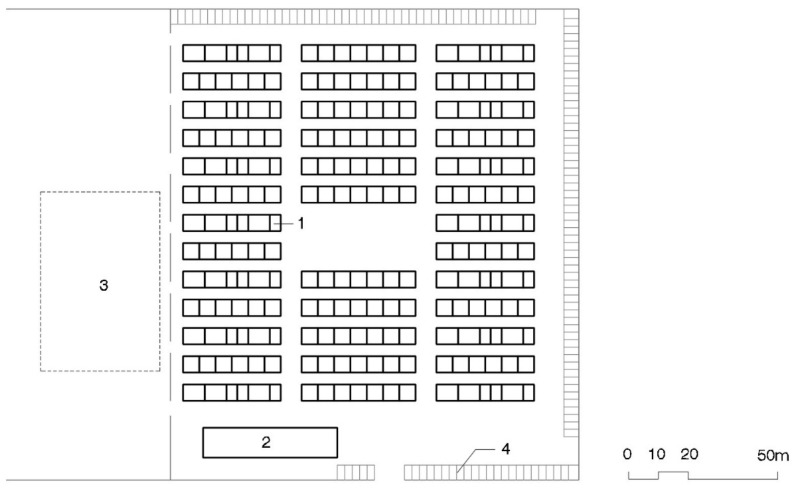
Plan of Tohoku’s 233 THA. 1—Temporary housing row; 2—Community Center; 3—Playground; 4—Parking area [48].

The number of temporary houses in each THA varied significantly due to the magnitude and extension of the disaster. In rural areas THAs are smaller and hold from 50 to 60 houses, while in cities, THAs can hold up to 250, like in Sendai, where eight THAs held 1505 houses ([23], p. 17). The total surface area of the THAs was kept at 100 m^2^ per house. The design of these areas was very similar to Kobe’s and Chūetsu’s (matrix of rows of six or seven houses) and includes the improvements implemented in 2004: a community center for every 50 THs and in some cases other installations to care for the most vulnerable residents ([20], p. 4.36). The location of the facilities, outside the housing area, and the parking, inside, was also similar (see Figure 6).

#### 3.3.3. Kodokushi

In Tōhoku 230 cases of *kodokushi* were recorded in six years (2011 to 2016), according to the National Police Agency, 97 in the first three years. Although the data is incomplete since statistics for six municipalities, Otsuchi and Miyako in Iwate, Kesennuma and Higashimatsushima in Miyagi, and Kawamata and Kawauchi in Fukushima, were unavailable [31].

### 3.4. Kumamoto 2016

The Japan Meteorological Agency named a sequence of more than 1700 discernible earthquakes from 14 April to 13 June 2016 the Kumamoto Earthquake. The three main earthquakes took place on 14–16 April. The first two had a magnitude over 6, and the third, the largest of the three, hit 7.3 [49]. It caused more than 49 deaths, destroyed more than 2800 and partially destroyed more than 5300 houses [50].

#### 3.4.1. Management and Organization

This is a very recent disaster, so the data is still being collected and there is practically no literature on the topic. The earthquake, despite its name, affected the prefecture of Oita as well as Kumamoto. The two prefectures were in charge of building 4300 temporary houses, both prefab units and conventional, as temporary wooden housing, thanks to the positive reception the wooden THs had in Tōhoku. Temporary housing was also offered for those affected who had restricted mobility [51] (p. 30). Some of the TH units were prepared in two months [52], but that was not the norm, and there were general delays to occupy the THAs due in part to the difficulties encountered to find publicly-owned land and to trouble insuring those lands (42 of the 132 sites initially proposed by the municipalities affected were in areas susceptible to flooding) [53]. At the end of 2018, more than 70% of the TH were still in use [54].

The delays opening most of the THAs meant that fewer spaces were initially offered to affected people in emergency camps than those requested (1300 applications for only 976 units). For this reason, in Kumamoto a lottery was also used to enter [52].

The mentioned delays also pushed through provisional housing alternatives other than THAs. By the end of March 2017, a total of 4303 temporary houses had been built, accommodating around 11,000 affected people. The prefecture of Kumamoto had rented at that time around 14,700 houses, which housed approximately 34,000 people, and used 1300 public housing and national civil servant lodging units to accommodate another approximately 3000 people ([51], pp. 29–30).

The community centers of Chūetsu and Tōhoku were maintained in Kumamoto. Activities were also organized by the local authorities and NGOs: the Nippon Foundation ran surveys to identify needs and then activities to support them [55]; the NGO Peace Winds organized workshops among residents and activities with pets [56]; and the NGO Recruit Sumai Company developed the “Green Curtain” project which consisted of planting vine plants to cover the windows of the TH units and reduce heat inside in the summer months at up to 270 TH units in Mashiro Town [57].

The “Home for All” project that Toyo Ito developed in Tōhoku was continued in the THAs in Kumamoto but not in an exceptional way like in Sendai: this time 80 meeting buildings were built in the different THAs [53], adapted to the cultural traditions and needs of each of the affected communities [58].

#### 3.4.2. Design

The construction quality of the light metallic structure prefab houses installed after the Kumamoto earthquake was better thanks to the lessons learned in temporary housing after the floods of 12 July 2012 which affected the city of Aso, Kumamoto. After the flooding, 48 temporary wooden houses were assembled whose thermal and acoustic insulation was improved beyond the usual standards. The standards of the prefab housing were revised when installed after the 2016 earthquake, demanding they be as similar as possible to those made of wood installed in 2012. The result was an improvement in the building quality (in terms of habitability and sound-insulating) of the prefabs, better than the TH of Tōhoku [53].

The models, sizes, and types of houses in Kumamoto were the same as those offered in Chūetsu and Tōhoku: one (20 m^2^), two (30 m^2^), and three bedroom (40 m^2^). There were a few notable exceptions, like the houses presented by Sigheru Ban (Pritzker Architecture Prize in 2014) in the Mifune, Kumamoto THA, which received the Good Design Award [59]. Here the layout, but not the size, of the houses, prefab and wooden, changed slightly by placing the closets as a separator (and noise insulation) between houses.

**Figure 7:**
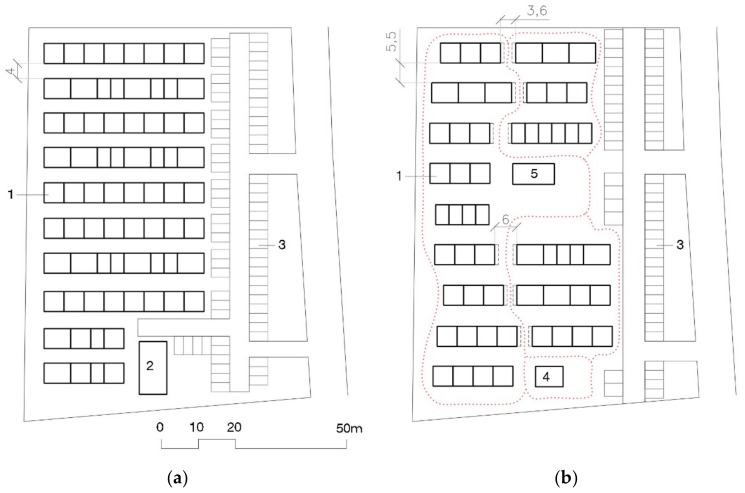
Kumamoto’s THA: (**a**) Plan of rejected 72 THA proposal (100 m^2^ × TH unit), 1-Temporary housing row; 2-Community Center; 3-Parking area; (**b**) Plan of modified 55 THA proposal (150 m^2^ × TH unit), 4-Common room (40 m^2^), 5-Meeting place (60 m^2^) [62].

The number of temporary houses in each THA was relatively small (50–60) with a few notable exceptions, like the Mashiro THA, with 516 THs [57]. The THAs in Kumamoto were larger thanks to the intervention of the architect Toyo Ito. This architect, Pritzker Architecture Prize winner in 2013, who had already worked on the Tōhoku THAs and launched the “Home for All” project, offered his collaboration to the governor of the Kumamoto Prefecture, Ikuo Kabashima, who accepted [60]. The JPAs’ first proposals for the THAs in Kumamoto, the same as in Chūetsu and Tōhoku, were rejected and modified with Ito’s proposals: the THAs’ surface area was expanded, from the 100 m^2^ of the usual THs to 150 m^2^; the separation between the rows of prefabs was increased from the standard 4 m to 5.5 or 6.5 m; and the rows were interrupted to facilitate perpendicular routes to them (see Figure 7) [53].

#### 3.4.3. Kodokushi

A total of 28 cases were recorded in three years in all of the provisional housing. Of these 22 were in rentals and only six in THAs [61].

## 4. Results

The Kobe and Tōhoku earthquakes destroyed more than 100,000 and 140,000 houses, respectively, and in both cases nearly 50,000 temporary houses were built. The Chūetsu and Kumamoto earthquakes destroyed around 3000 houses, and in the first more than 3000 houses were built and in the second more than 4000. Japanese disaster management specifies the need to learn for the next large-scale disaster, tacitly distinguishing between small, medium, and large-scale disasters [51,63,64]. The results and conclusions derived from managing the disasters of Chūetsu and Kumamoto should always be sifted through the optics of their scale. In the case of Kumamoto, we must be especially prudent in this section and in the conclusions, since the disaster is still very recent and in its recovery stages.

In the case of Kobe, there is a direct relationship between the high number of lonely deaths (around 250 cases) and the breaking of community ties that predated the disaster. The distance of the THAs from the affected areas broke the ties between residents and their heretofore known environment and the lack of prior relationships between the THA residents, a consequence of choosing an arbitrary entry system (lottery), tore apart the relationships neighbors had before the disaster ([26], IV-8; [18], p. 200; [29], p. 59; [30], p. 36). For this reason, after 1995 Kobe, *kodokushi* has conditioned and guided changes in housing program management policies.

At Chūetsu and Kumamoto, small and medium scale earthquakes that affected wide swathes of land with less intensity, the THAs could be placed near the affected areas. However, in Tōhoku, due to the complexity of the disaster, some of the THAs of the people from the flooded areas and of those evacuated because of radiation were far from their original houses.

The lottery entry system in Kobe’s THAs was corrected in Chūetsu, where maintaining original communities was prioritized and achieved. In Tōhoku, community-based THAs were also prioritized. However, the disaster’s complexity meant it was not always possible, and the lottery entry system was used at the beginning when demand was highest ([20], p. 4.36). In some cases, like in Minamisanrikku, Miyagi, up to 62% of residents entered through this system ([18], p. 198). Even in an earthquake of a very different scale, like Kumamoto, the lottery entry system was used again.

In Kobe’s provisional housing supply, temporary housing was practically 100%. After Chūetsu the supply of provisional housing increased, including private rentals and public housing. However, these alternatives were rare and symbolic only. This was not the case of Tōhoku, where temporary housing was only 35% of the total of occupied provisional housing. In Kumamoto this percentage shrank even more, to 25%.

In Chūetsu, for the first time services to attend to residents were implemented to reduce or eliminate isolation. In Tōhoku and in Kumamoto, those services were kept inside the THAs. The NGO and volunteer association initiatives (services and projects) grew to reduce resident isolation.

The construction quality of prefab housing in Kobe THAs was very low. In Chūetsu, as part of the lessons learned from Kobe, improvements were made, thanks to the inclusion of thermal insulation. However, in Tōhoku, minimum quality standards were used again for the light metallic structure prefab houses. In Kumamoto, the building quality increased substantially and the standards were improved, including thermal insulation and, for the first time, sound insulation.

Some of the problems that appeared are recurring and inherent to the light prefab housing with very little thermal inertia, which results in condensation and mildew, as well as noise transmission problems. In Chūetsu, incorporating insulation and air conditioning improved habitability but did not prevent condensation. In Tōhoku, the main prefab TH building issues also stemmed from the lack or limited use of thermal insulation given the harsh winters in affected areas ([18], p. 196). Although attempts were made to alleviate Tōhoku through supporting initiatives from local companies and NGOs by including insulation or soundproofing materials the basic construction problems remained in most cases ([18], p. 198).

The second type of problem has to do with deficient assembly which tends to provoke gaps between walls and roofs and as a consequence mildew from water coming in, lack of thermal comfort inside, etc. This second type of problem is related to the scale of the disaster. The enormous demand for TH units generated after a mega-disaster means specialized companies have to manufacture many more units in the same time as in a small disaster. Those companies are incapable of covering all the demand so non-specialized companies start to manufacture THs, which sparks a drop in required quality.

Wooden houses deserve a special mention. In Tōhoku, the three prefectures built temporary wooden houses. There was a large variety of kinds but in general they were well received and at a price quite similar to prefab houses’. One of the problems was they took slightly longer to manufacture ([20], p. 4.36). There was also a problem with recycling, since prefabricated temporary housing, with years of experience, has a reuse system that the wooden units lack [46].

From the point of view of layout, the size and number of types offered substantially improved between 1995 Kobe and 2004 Chūetsu, as can be seen in Figure 1 and Figure 3. In Kobe, families of four or five had to live in a space of 26 m^2^. In Chūetsu, three models were offered: 20 m^2^, 30 m^2^, and 40 m^2^. In Tōhoku and in Kumamoto the same models were repeated, with the same layout and the same sizes (including non-prefab varieties, like those of wood). Increasing the house’s façade was also an improvement in the distribution since it eliminated rooms without natural light and was an improvement from the point of view of hygiene (ventilation and accommodations): from the 3.6 m of the two Kobe models, to the 3.6, 5.4, and 7.2 m for the one, two, and three bedroom models in Chūetsu, Tōhoku, and Kumamoto.

THA layout has barely changed since 1995 Kobe, as can be seen in Figure 2, Figure 4 and Figure 6. Houses are placed in rows of between six and ten (in Kobe longer, ten, than in Tōhoku, six or seven). These groups, always facing the same direction, form a matrix in which parallel rows, four meters apart from each other, make streets to access the houses. Streets six to eight meters wide are formed perpendicularly to the rows. Sometimes a square is added by eliminating a pair of rows: this is the most like a public space that can be found in these THAs. Parking in rows for resident vehicles is on the perimeter of the camp.

The only major variation to this scheme was in Kumamoto where, at Toyo Ito’s request, the length of the rows was cut back, to two, three, and at most four houses, to create cross routes to them. The THA surface area was also increased, from 100 m^2^ per house in Kobe, Chūetsu, and Tōhoku, to 150 m^2^ per house.

THA facilities and services improved after Chūetsu by including a community center for every 50 TH units and sometimes specific facilities for the most vulnerable people. These facilities have not changed the matrix of TH rows described, not in Chūetsu, not in Tōhoku, and not in Kumamoto, but have been placed on their outside perimeter or by substituting one of the rows.

No cases of *kodokushi* were recorded in Chūetsu during the three years the THAs were in use. In Tōhoku, there were 230 cases of *kodokushi* in six years. Six cases have been recorded in the Kumamoto THAs in the first three years.

## 5. Discussion

Since Kobe 1995, *kodokushi*, or lonely death, has conditioned and steered changes in THA management policies: an attempt was made for THAs to be placed close to the affected neighbors’ original houses and to keep the original communities together in the same THA, avoiding the lottery system. However, these two strategies together could only be applied in a small scale disaster like in Chūetsu, where the lack of cases of *kodokushi* confirms that the recovery processes using community-based strategies improve the process’ resilience by taking advantage of “the power of place” ([65], p. 347). It is unquestionable that maintaining the same community in the same THA close to the disaster naturally creates resilient communities and with more self-organization capacity–bottom-up approach ([66], p. 721).

However, we have seen how in a large disaster like Tōhoku these two simultaneous strategies could not be carried out in many cases, which led to more vulnerable and dependent communities. Local governments, in collaboration with NGOs and private companies, have tried to reinforce these communities, especially the most vulnerable groups, in different ways: through medical and psychological care or through different kinds of services (food, baths, etc.) or by organizing activities to build links between residents. After Kobe, the profiles of the people that have more risk to suffer kodokushi are known: firtsly, there are residents that live alone, low income and jobless ([18], p. 212); secondly, the risk is 2.3 times higher among men than among women; finally, kodokushi is related to alcoholism ([26], IV-8). Social services inside the THAs know these profiles. However, the current top-down approach, based on supporting measures, has caused problems among residents. The efforts of volunteers and medical care services to check on the health of residents with continual visits to the THs led to complaints because they were stressful and invaded their privacy. This gave rise to a curious protocol: those residents who did not want to be bothered were to place a yellow flag each morning on the door of their residence ([32], p. 168). It was also confirmed that activities organized by volunteers, NGOs, and other groups did not lead to significant changes in habits for the most vulnerable groups in terms of loneliness and isolation [23,32].

It should be pointed out that architecture (facilities, size, types, and layout of the THs) and urban design (distribution of THAs, their public-to-private space ratio, their facilities, etc.) condition and promote different lifestyles. However, and despite the fact that since Kobe we know that there are problems that fall within the field of design, architecture, and urban development, very few changes have been made from these points of view.

The facilities in Japanese temporary housing have barely changed in the last 25 years. The THs after the 1993 southwest-off Hokkaidō earthquake already had dining rooms, kitchens, bathrooms, and toilets ([20], p. 3.10), like the models installed in the four disasters we have analyzed. However, it is clear that a house of this type allows and encourages a type of autonomous and self-sufficient life inside of it, since all of the residents’ daily activities and routines can be carried out inside the house.

Smith [32] has compared Tōhoku’s THAs to Sichuan’s (after the 2008 earthquake more than a million THs were built). The Chinese THs consisted of a single bedroom with no bath or running water, so the THAs had common dining rooms and bathrooms. Smith argues that the fact that some of the daily activities had to be done outside of the TH units, by forcing daily contact, ended up increasing socialization and the creation of ties, and in part—beyond the cultural and personality differences between the Chinese and Japanese—explain the lack of cases of *kodokushi* in Chinese THAs.

Since Chūetsu, there have not been any changes to the types offered (three models), the sizes (20, 30, and 40 m^2^), or the layout inside. There were no changes made although the temporary housing had been planned years in advance, like with some of the Sendai models that were planned eight years before Tōhoku 2011 ([67], p. 182). The planning “only” served to improve the TH’s technocratic side: it reduced the THA implementation to less than a month and a half but did not affect design. Nor has the participation of construction companies not part of the JPA changed facilities, types, or layout. The change in building system and material did not lead to changes either: the wooden THs are fundamentally the same with only minimum variations [68,69]. Even the models proposed by renowned architects like the 2013 and 2014 Pritzer Award winners Toyo Ito and Shigeru Ban maintain facilities and types and only introduce minor changes to layouts.

There were no major urban design changes (THA design and layout) either. The Kobe, Chūetsu, Tōhoku, and Kumamoto THA ground plans (see Figure 2, Figure 4, Figure 6 and Figure 7) have the same pattern and aerial photographs of the THAs are indistinguishable. Japanese THAs are created based on the sum total of self-sufficient THs. There is practically no concession to something that looks like a public space. The matrix layout of the houses, all facing the same direction, means entry doorways never face each other and reduces the possibilities of interaction between neighbors.

The pattern used is so basic that, due to the Kumamoto disaster, the prefab temporary housing company Daiwa and the Kumamoto University signed a deal to be able to obtain the ground plan of a THA in an hour [70]. These types of actions are typical of (unplanned) after-the-fact disaster management that lays the emphasis on the universality of the solutions, speed of design and execution (technocratic approach), and not on the end user (human-centered approach).

Japanese THAs were not, however, always that way. The camps set up in Tokyo in public parks after the 1923 Great Kanto earthquake had several facilities: public spaces for bathing, bathrooms, and kitchens, and others like medical clinics, nursery rooms, informational offices, bookstores, stores, beauty salons, etc. ([20], p. 3.4; [23], p. 4). The accommodations were barracks with no type of services, which forced residents to carry out significant parts of their daily lives in public areas.

In the period covered by this study–which covers the four disasters analyzed–there are individual or specific solutions that pose very interesting amendments and proposals. In Kobe, 5000 self-build TH units were built ([20], p. 3.29). Despite of these housings had not been funding by the government–because they were assembled on private plots-these neighbors preferred to remain near the affected area in order to keep in contact with their community, their neighborhood and their stores and businesses ([29], p. 60). This kind of initiatives referred as “recovery *machizukuri*”—term appeared in the 1950s for describing neighborhood initiatives led by citizens ([21], p. 22)—has had institutional support by the Architecture Institute of Japan–one of the biggest academies of the country ([22], p. 239). In Chuëtsu, the aforementioned project “Temporary Open Café” developed by several researchers and professors from different Japanese universities proposed resilient activities among the residents of the THAs [30]. In Tohoku, the initiatives of different associations to improve resident’s life within the THAs were multiplied. Some of them were gathered in the “How Did Architects Respond Inmediatlely after 3/11 The Great East Japan Earthquake” global travelling exhibition [71]. Several organizations, 15 university laboratories and with the support of the Architecture Institute of Japan, exhibited over 50 projects, all of them with an humanistic approach, for example: the proposals of the group Kishin (yearning for a home, in Japanese language), the group ArchiAid or the Toyo Ito project “Home for All”. With respect to the urban planning, in the rural and coastal areas of Tohoku, the Association of Rural Planning proposed bottom-up alternatives (in the *machizukuri’s* spirit) to the government-led reconstruction plan ([22], p. 240).

The Tokyo University suggests that THAs not be understood as the sum of individual houses but as a Temporary Town [72]. There is an example of the application of these recommendations after the 2011 disaster: the Temporary Township of the city of Kamaishi, in the prefecture of Iwate. It is an isolated project which was ideated by the Tokyo University [73]. A temporary shopping mall and 240 temporary houses are placed together in the same space. The mall has 22 tenants, with a café, beauty salon, a small supermarket, etc. The initiative was a success and was used as a lesson for future disasters ([20], p. 7.19).

An interesting contribution in this field is the THA of Mifunetown, Kumamoto. There the reduced length of the rows to improve cross connections proposed by Toyo Ito combined with the special housing solution proposed by Shigeru Ban, which designs public baths in the new spaces between rows, improved public spaces and the relationship areas in the THAs. The combination of these two talents provided one of the best solutions of the Japanese THAs, although it remains an exceptional and unique one.

Nevertheless, some scholars maintain that only machizukuri and bottom-up approaches are not enough for solving the complex problems related with Disaster Risk Reduction (DRR). In their opinion, urban planning, civil engineering and architecture cannot respond solely to disasters ([22], pp. 239–240), and they stand up for an interdisciplinary approach. This is the case of the Japan Academic Network for Disaster Reduction [74], that groups more than 40 academic societies for integrating different specialties for disaster reduction. Based on the lessons learned from 2011 Tohoku earthquake, they defend to develop “mutual understanding and made efforts to integrate different specialties” [75].

Perhaps due to the lack of changes to the Japanese THA design, in the last few years a noticeable shift has occurred in provisional housing offered in Japan. The percentage of TH offered was cut back from practically 100% in Kobe and Chūetsu to 35% in Tōhoku and a mere 25% in Kumamoto. This is understandable since private rental apartments have their advantages: their cost is much lower, on average $9000 per year or $18,000 for two. The cost is between $71,000 and $80,000 per unit for prefab houses in Tōhoku. The price is paid directly by the Japanese government and those affected can enter immediately after the disaster without the THA wait times (from a month and a half to six months). In addition, residents consider them more comfortable ([18], p. 197).

That said, they cannot always be a solution because in a large-scale disaster the magnitude of the destruction in some of the areas affected either reduces or eliminates existing stock. There is also another problem that until now has not been studied sufficiently: the invisibility and isolation of those who occupy them ([18], p. 197). Scattering the people affected breaks their bonds with their previous communities, which, as we have seen, is one of the major sources of problems related to residents’ mental health. In fact, a health survey recently published of the disaster survivors of the Great East Japan Earthquake and Tsunami [76], conclude that THA’ residents, thanks to larger social participation than those of rented housings, have larger health benefits.

## 6. Future Lines of Work and Recommendations

**Figure 8:**
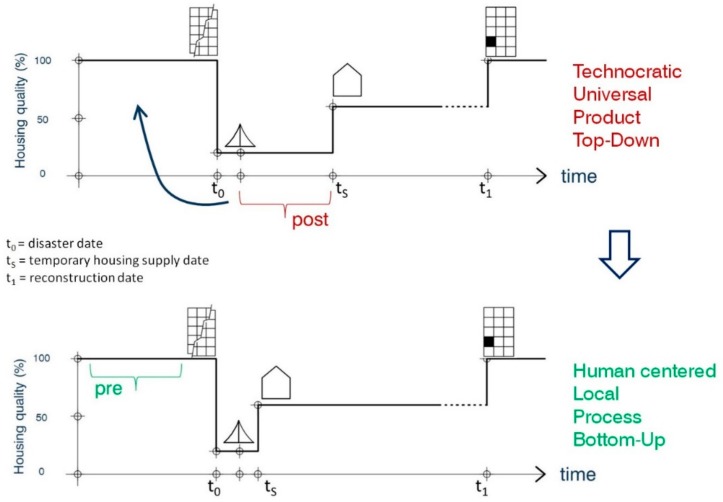
Changing the planning of THA from post-disaster time to pre-disaster time.

Japan is one of the only examples in the world in which the design of temporary housing was planned in advance [67], in Sendai. However this planning was only used to improve the building, installation, and assembly process. The advantages of working in advance, precluding the chaotic situations after a disaster, were only leveraged from a technocratic point of view. In this paper we propose that Japan work in advance to improve the THA from other perspectives, going beyond the technocratic view (see Figure 8).

### 6.1. From a Technocratic Approach to a Human-Centered Approach

Till now, THAs have been approached as engineering problems and engineering problems alone. As Nelson and Stolterman state ([77], p. 210): “a needs-based change, animated through a problem-solving approach, assumes that the right outcome is known from the start”. And, in effect, the specialized literature [6,7,8,9,10] and Japanese disaster management [36] reduce the needs of affected people to being supplied with a house. But a house is not a need; it is a solution to certain needs. The role of the THAs is not clearly defined, and as Davis and Alexander point out ([67], p. 192), the functions listed in 1982 ([4], p. 8) of providing safety and privacy, and in general to allow affected people to return to their normal lives, have not been changed or revised since then. This generic and abstract vision (back to normal) is also the one that appears in the disaster management manuals in Japan ([64], p. 66).

Applying a design methodology that opens the way to innovation in planning, project, and development of the THAs seems recommendable. For one, it would expand the vision of what the affected people’s needs are, including their desires and wishes which will be basic in recovery: “people desire to flourish and not just to survive” ([77], p. 210). For another, based on the needs and desires that must be covered, it would open the door to innovative solutions that go beyond physically supplying a house. Maybe affected people should be provided with a home before a house. According to Pallasmaa [78], it is the loss of home which triggers and is synonymous with exclusion, loneliness, and a perpetual present indicative, that is, the lack of a future. Although the difference and relationships between the concept of house and home have been analyzed in the past [79,80,81], its application to the field of temporary housing might be extremely useful, given that psychological, loneliness, and isolation problems–that could potentially lead to *kodokushi*—have more to do with the idea of the loss of home—more related to the “psychology, psychoanalysis and sociology” ([78], p. 2)—than with the physical loss of a house or living space.

The philosopher Takashi Uchiyama criticized the Japanese government’s lack of clear goals during it management of the Tōhoku disaster ([22], p. 238). From his perspective, “recovery is neither revival economy, nor rebuilding houses”; what in his opinion should characterize the recovery process is the rebuilding of relationships between people and between human beings and nature. This vision, much more humanist and focused on people, could be an example of how the needs of affected person and the role of the THAs could be reframed.

### 6.2. From Global to Local

Prior planning would allow adapting THAs to the characteristics of the different areas in Japan, not just from a building perspective but also from a cultural one. The positive reception of the wooden models by people affected from Tōhoku and Kumamoto was especially significant. However, from our perspective, the problem is not prefabrication per se, because to respond to the high demand for temporary housing of a large-scale disaster there is no better option [6], since it allows for fast manufacturing, supply, assembly, and disassembly [82]. In fact, there are examples in Kumamato of temporary housing prefabricated in wood: Shigeru Ban’s recently awarded project in Mifune [52]. However, this is an outlying proposal. Most of Japanese prefabricated houses have not leveraged enough on the current customization possibilities afforded by prefabrication [83,84].

### 6.3. From Product, TH Module, to Process, THA

The way people live is not exclusively determined by the private sphere. In post-disaster times, it is important that residents of temporary housing stay physically and socially active, since one of the major problems in the post-disaster period is psychological, which intensifies with isolation and loneliness. The design of the THAs conditions its residents’ lifestyle, so the THA cannot be understood exclusively as the sum total of the temporary housing units but as a comprehensive design of the entire area, determining which daily activities will be private and which will be public. It is the aggregate of the THA which must cover the affected people’s needs. This wider and comprehensive concept of the THA has been pointed out and defined with different names before: second city [85] or temporary town [72]. The vision of the THA as a part of a process in the total recovery increases if its design is integrated into an urban design plan. The THA’s design can be taken as a chance to improve the city: the connection between urban design and recovery would guarantee a resilient and sustainable city.

### 6.4. From a Top-Down to Bottom-up Approach: Community Participation

**Figure 9:**
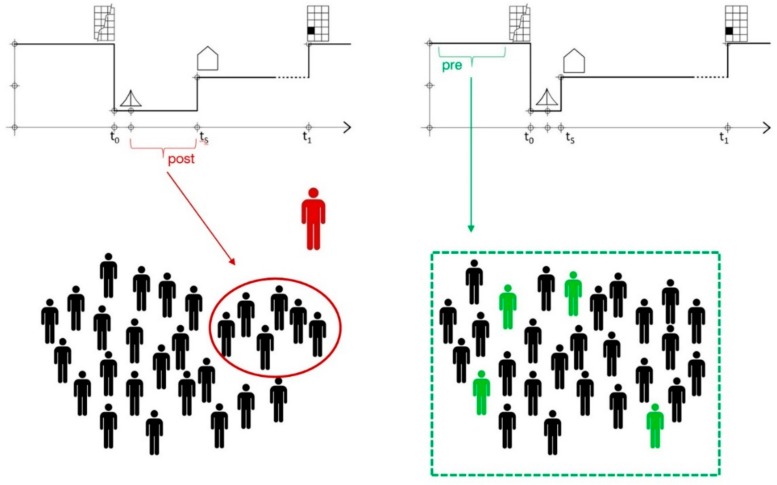
Post-disaster and Top-Down approach vs. Pre-disaster and Bottom-Up approach.

Participatory processes in the scope of temporary housing are not new. They have been held on a number of occasion but always after the disaster [86]. However, the chaotic conditions after a disaster are not the best in which to launch a participatory process. The lack of planning prior to the disaster in the field of provisional housing leads to highly centralized processes led by the government, or a top-down approach. As argued by Puliafito [87], “democracy is not possible in emergency”; there are very few stakeholders who participate in management, generally specialist professionals and politicians. With these kinds of mechanisms, individuals, communities, and even city councils are pushed to the sidelines in planning. This leads to a paternalist and charity-based approach. Moving planning of temporary housing to pre-disaster time would allow involving other important stakeholders with a more humanist profile like architects, designers, urban planners, cultural associations, etc., and launching a citizen participatory process in temporary housing (see Figure 9).

## 7. Conclusions

The design of Japanese temporary housing has hardly changed in the last 25 years, since 1993. The modules or houses are conceived as small self-sufficient apartments that include all the facilities necessary for residents to lead a self-sufficient and independent life inside them. The THAs are the result of the sum total of these small apartments, which are laid out in a regular, repetitive, and undifferentiated way to optimize available space, without making practically any concessions to public or interrelation spaces. With this structure the goal was to respond in the fastest, most effective, and most feasible way, using technocratic criteria, to the explicit demand in the management of Japanese disasters to supply a house to those affected that cannot pay for one ([36], art. 23). The idea that implicitly underlines this approach is that the needs of affected people after a disaster are the same as they had before: if they lost a house, they need a house.

An approach more focused on human beings, and on the real needs of the affected people, shows that after a disaster affected people have not only lost their houses but also their home, all their belongings, which provided them with their identity beyond the merely material realm. In many cases, the loss of family members and friends is added to this. Losing homes and identity also triggers exclusion, loneliness, and the feeling of a lack of a future. After a disaster, the risk of social health problems is added to physical and mental problems: the relationships that the affected people had with friends, neighbors, and acquaintances in their communities are broken. But it also entails the risk of breaking ties with their environment: the services and facilities network (stores, health care centers, libraries, etc.) that affected people used before the disaster.

The Kobe disaster showed in sharp relief the effects of a design that promoted independence and self-sufficiency but also isolation and was undifferentiated and universal, culturally foreign to the original wooden houses. The combination of this design with THAs located far from the affected areas (breaking ties with their setting) and a random entry system (which wiped out the affected people’s prior relationships with their communities) lead to the groups most vulnerable to mental health problems remaining in isolated situations in which it was very hard to help them, which at its most extreme led to *kodokushi*.

The disaster in Chūetsu showed that applying appropriate management policies to ensure social health (THAs close to affected areas and keeping pre-disaster communities together in the THAs) in combination with the medical and psychological support services and other activities to improve community bonds (held in community centers created for that purpose) was able to offset the isolation and loneliness that the THA design fostered. These measures, combined with the disaster’s small scale, thanks to which the management measures could be applied, and the short period of time the camps were used, three years, improved the mental health of residents: there were no cases of *kodokushi*.

The disaster of Tōhoku has shown that in a large-scale disaster guaranteeing the simultaneous application of management measures designed to guarantee social health is not possible. Sometimes safe plots near the affected areas cannot be found, others, preexisting communities cannot be moved to the same THAs. Although in Tōhoku the medical support services and the activities to improve community bonds were increased, these measures were not enough to alleviate the mental problems of some of the most vulnerable residents, as they were aggravated by social health problems.

Do people made homeless by a disaster have the same needs before and after? Japanese disaster management which, as we have seen, dedicates enormous management, organization, and services efforts to improving the mental and social health of those affected knows the answer all too well. If the needs then are not the same, why respond with the same housing and THA design? The basic conclusion of this study is that the design of Japanese THAs is promoting a lifestyle which does not respond to the real needs of the most vulnerable groups after a disaster.

It goes without saying that it is always advisable to apply management measures whose goal is to improve social health (setting up THAs near affected areas and community-based THAs) but the reality has shown that in large-scale disaster as complex as Tōhoku they cannot always be implemented. In those cases, we have seen how services to alleviate residents’ mental problems and the activities organized inside the THAs that attempted to strengthen the bonds between residents–and by doing so their social health–were insufficient. These services and activities have attempted to correct the problems caused by architecture and design.

Efforts have been made in recent times to improve the design of THAs, the most significant of which was Toyo Ito’s contribution to Kumamoto. His post-disaster contribution improved public spaces moderately but did not extend to housing and in our opinion does not cover the affected people’s real needs because it focuses on increasing THA meterage and not on reducing their isolation.

Because architecture and design should be part of the solution and not the problem, this paper concludes that the THAs’ design should be reconsidered to make sure they cover the homeless people’s real needs. A project of this caliber should be planned, starting pre-disaster (far from the chaotic conditions post-disaster), dedicating the necessary time, probably years (and not drawing up THA ground plans in an hour), using a suitable team (possibly a multidisciplinary team that includes humanist backgrounds, preferably of the likes of Toyo Ito and Shigeru Ban), specifically technical backgrounds (engineers from different disciplines), manufacturing companies, etc. A team of that caliber could design a THA model that generally included these approaches, considerations, and visions that until now have not been considered or have only been developed on rare occasions as isolated examples of Japanese THAs: the definition of the real needs and desires of the homeless; the adaptation of the design of the housing to the Japanese local and cultural reality (wood as a material is only one aspect of this); a comprehensive design of the THA, studying in detail which activities should be private and which public, understanding the ensemble not as the sum of its parts but as a process, a total of services to cover the needs of the affected people in the recovery stages; the inclusion of mechanisms to ensure community participation in the process; and, lastly, the conception of the THA as an active and resilient tool in which residents feel they are part of the process, ultimately, understanding THAs as springboards to recovery.
